# Characterization of Acetonitrile-Tolerant Marine Bacterium *Exiguobacterium* sp. SBH81 and Its Tolerance Mechanism

**DOI:** 10.1264/jsme2.ME11228

**Published:** 2011-10-05

**Authors:** Ajiraporn Kongpol, Junichi Kato, Takahisa Tajima, Alisa S. Vangnai

**Affiliations:** 1 Graduate Program in Biotechnology, Faculty of Science, Chulalongkorn University, Bangkok 10330, Thailand; 2 Department of Molecular Biotechnology, Graduate School of Advanced Sciences of Matter, Hiroshima University, Hiroshima 739–8530, Japan; 3 Department of Biochemistry, Faculty of Science, Chulalongkorn University, Bangkok 10330, Thailand; 4 National Center of Excellence for Environmental and Hazardous Waste Management (NCE-EHWM), Chulalongkorn University, Bangkok 10330 Thailand

**Keywords:** organic-solvent tolerant bacteria, organic-solvent tolerant mechanisms, cell adaptation, *Exiguobacterium*

## Abstract

A Gram-positive marine bacterium, *Exiguobacterium* sp. SBH81, was isolated as a hydrophilic organic-solvent tolerant bacterium, and exhibited high tolerance to various types of toxic hydrophilic organic solvents, including acetonitrile, at relatively high concentrations (up to 6% [v/v]) under the growing conditions. Investigation of its tolerance mechanisms illustrated that it does not rely on solvent inactivation processes or modification of cell surface characteristics, but rather, increase of the cell size lowers solvent partitioning into cells and the extrusion of solvents through the efflux system. A test using efflux pump inhibitors suggested that secondary transporters, *i.e.* resistance nodulation cell division (RND) and the multidrug and toxic compound extrusion (MATE) family, are involved in acetonitrile tolerance in this strain. In addition, its acetonitrile tolerance ability could be stably and significantly enhanced by repetitive growth in the presence of toxic acetonitrile. The marked acetonitrile tolerance of *Exiguobacterium* sp. SBH81 indicates its potential use as a host for biotechnological fermentation processes as well as bioremediation.

Organic solvents (referred to as solvent) can be classified into two groups: hydrophilic and hydrophobic. Hydrophilic solvents, such as acetonitrile, have been extensively used in analytical settings and as solubilizing agents in the plastic and petrochemical industries. Recently, their roles in biotechnological and pharmaceutical industries have been increasing. For example, they have been involved in biocatalysis-based production as a co-substrate, biological media or product (23); however, even at low concentrations, solvents have destructive effects on microbial biocatalysts due to their toxicity. Therefore, bacteria with solvent tolerance characteristics have been explored for their potential use in biotechnological production and for industrial waste treatment where solvents exist at high concentration.

Organic solvent-tolerant (OST) bacteria are a group of extremophilic microorganisms that can thrive in the presence of a high concentration of solvent as a result of their indigenous tolerance mechanism (19). Because of their advantages, studies have been conducted to screen, isolate and characterize novel OST bacteria, to investigate solvent tolerance responses and mechanisms (14), or to improve tolerance by adaptation, genetic engineering or metabolic engineering (4). Numerous reports have investigated Gram-negative OST bacteria, especially *Pseudomonas* and *E. coli*, and focus particularly on their tolerance to hydrophobic solvents; however, there are only a few reports on Gram-positive OST bacteria, such as *Bacillus* sp., *Brevibacillus* sp., and *Rhodococcus* sp. (7, 12). As the exploitation of hydrophilic solvents has been continually increasing, demands on bacterial catalysts able to tolerate such solvents are expected. Nevertheless, hydrophilic solvents with high polarity and a low log *P_ow_* value (<1) have high toxicity to cells; therefore, finding such bacteria has been a challenge (23).

This study aimed to isolate and characterize bacteria able to tolerate hydrophilic solvents at high concentration. As a result, a Gram-positive marine bacterium, *Exiguobacterium* sp. SBH81, with unique tolerance ability to acetonitrile is reported and its tolerance mechanism is postulated. This report is the first to describe a marine bacterium able to tolerate high concentrations of acetonitrile under culturing conditions.

## Materials and Methods

### Chemicals and cultivation medium

The solvents in Table 1 were from Fluka (Steinheim, Germany). The efflux inhibitors, orthovanadate, paroxitine and PAβN, were from NacalaiTesque (Kyoto, Japan), Toronto Research Chemicals (North York, Canada) and Sigma (Kanagawa, Japan), respectively. The culture medium components were from NacalaiTesque. Bacterial cultivation medium was either Luria-Bertani (LB) medium or minimal salt basal medium (MSB). When it was supplemented with yeast extract (0.1% [w/v]) and glucose (3.5 gL^−1^), it was abbreviated as MSBYG.

### Isolation, identification and characterization of hydrophilic solvent-tolerant bacteria

Bacteria were screened from seawater samples collected from the Gulf of Thailand, Petchburi province, Southern part of Thailand. Seawater samples were mixed with LB medium and incubated at room temperature (~33°C) for 8 h. *n*-Butanol was provided at 0.1% (v/v) and incubated with shaking overnight. The culture was then diluted and plated onto LB medium agar to obtain single colonies. Isolates with different colony morphologies were selected and examined for their tolerance to hydrophilic solvents. An isolate with relative high tolerance to acetonitrile, which was the hydrophilic solvent of interest, was selected for further investigations.

The selected isolate was identified by a biochemical test (National Institute of Health, Thailand) and 16S rRNA sequence analysis (7). The partial 16S rRNA sequence (1,316 bp) was analyzed using BLASTN and submitted to the GenBank nucleotide sequence database (NCBI) with the accession number HQ901077. A phylogenetic tree was constructed using Mobyle@Pasteur v1.0 (Institut Pasteur, France). The generated tree was analyzed with TREEVIEW program 1.6.6 and its growth characteristics were determined under various conditions, including pH (4–13), temperature (5–60°C) and salinity (0–15% [w/v] NaCl). The strain was deposited in the Thailand Culture Collection (BIOTEC, Thailand) under biological material number BCC45740.

### Organic solvent tolerance and utilization

The solvent tolerance test was conducted by two methods. First, cells were grown in LB medium at 37°C to the mid-exponential phase before solvent was directly added at 1, 5 and 10% (v/v). After 12 h of solvent exposure, cell survival was determined as colony-forming units (CFU) per milliliter. Second, to test cell solvent tolerance under growing culture conditions, acetonitrile at various concentrations (1–10% [v/v]) was added simultaneously with bacterial inoculum in MSBYG medium. Cell growth, determined as cell optical density at 600 nm (OD_600_), was used as a parameter for cell viability and tolerance. Cell ability to utilize acetonitrile for growth was determined by measuring the OD_600_ of cells grown in MSB medium supplemented with acetonitrile vapor.

### Cell adaptation to acetonitrile

Cells were grown in MSBYG medium supplemented with acetonitrile at 4% (v/v) at 37°C for 12 h (representing one cycle) and used as cell inoculum for the subsequent batch. The repetitive batch of bacterial cultivation with a constant level of acetonitrile was conducted for 10 and 30 cycles to obtain acetonitrile-adapted cells.

### Determination of acetonitrile tolerance mechanisms in Exiguobacterium sp. SBH81

Cell morphology was visualized using a scanning electron microscope (SEM) (JSM-5900; JEOL, Japan) after cell preparation (12). Cell dimensions were measured on SEM photographs to calculate surface area and volume (13).

Cell surface characteristics were examined using the bacterial adhesion to hydrocarbon (BATH) test (7). To test the formation of solvent-emulsifying agents, the spent supernatant of cells grown in MSBYG medium was vigorously mixed with *n*-tetradecane (5:1 [v/v]). Emulsion formation and stability was determined by measuring the height of the emulsion layer (if any) at 0 h and after 24 h and comparing with the control (fresh MSBYG medium).

Effect of efflux pump inhibitor (EPI) on acetonitrile tolerance was examined by growing cells at 37°C in MSB medium supplemented with each EPI: orthovanadate (0.1 and 10 mM), paroxitine (0.1 mM) and PAβN (0.1 mM) in the presence of acetonitrile vapor. Cell growth was monitored by measuring OD_600_.

## Results and Discussion

### Screening, isolation and identification of bacteria able to tolerate hydrophilic solvents

In this study, seawater was used as a source for screening hydrophilic OST bacteria because greater biodiversity has been reported in the marine environment than in soil (6, 19). Nine bacterial isolates obtained from screening were challenged with a range of hydrophilic solvents at a higher concentration (5% [v/v]). Among them, isolate SBH81 was selected for further investigation because it exhibited relatively good tolerance to a wide range of hydrophilic solvents (Table 1).

**Fig. 1:**
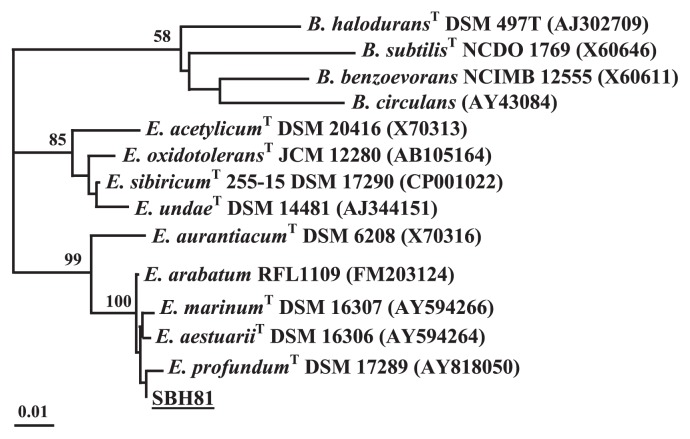
Phylogenetic relationships of *Exiguobacterium* strains based on 16S rRNA sequence comparison. The tree was generated using a neighbor-joining method with bootstrap values of 1,000 replicates. Scale bar infers 0.01 changes per nucleotide position. *Bacillus* strains were clustered in proximity as an outgroup. SBH81 (underlined) is the bacterial isolate obtained in this study. The NCBI GenBank accession numbers of bacterial type strains (^T^) and bacteria are shown in the parenthesis.

Isolate SBH81 is a Gram-positive, non spore-forming, rod-shaped, motile bacteria and forms orange-pigmented, spherical colonies on LB medium. It was identified by biochemical analysis as *Exiguobacterium acetylicum*. Analysis of a partial sequence of 16S rRNA indicated 100% sequence similarity with *Exiguobacterium* sp., *e.g. Exiguobacterium* sp. LY3 (EU073122.1), *Exiguobacterium* sp. WW12 (EF433553.1), whereas phylogenetic analysis showed that it is closely related to *E. profundum* (Fig. 1). In this study, we refer to this isolate as *Exiguobacterium* sp. SBH81 or SBH81.

### Growth characteristics and hydrophilic solvent tolerance of SBH81

The *Exiguobacterium* genus exhibits very diverse and unique cell properties, including psychrotrophic, mesophilic and moderate thermophilic, with morphological diversity and activity diversity (21); therefore, the growth characteristics and distinct properties of OST bacterium SBH81 were further investigated.

SBH81 could grow at a wide range of pH (5–12), temperature (20–50°C), and at high salt concentration (up to 10% [w/v]). Under optimal growth conditions at pH 8–9, 37–40°C, and 2–4% salinity, the growth rate was at 0.24±0.044 h^−1^. Although SBH81 was isolated under *n*-butanol-enriched conditions, it could tolerate *n*-butanol at only a moderate level, whereas it was highly tolerant to a range of highly toxic hydrophilic solvents, including acetonitrile, at high concentration (up to 10% [v/v]) (Table 1). Previous reports showed that *Exiguobacterium* strains have various unique properties, but there was only one report of *E. gaetbuli* TF-16 able to tolerate hydrophobic solvents such as toluene (10% [v/v]) and benzene (5% [v/v]) (22). The results showed that SBH81 possesses distinct and strain-specific hydrophilic solvent tolerance ability.

### Acetonitrile utilization and tolerance of SBH81

Among the hydrophilic solvents tested, cell tolerance to acetonitrile was of particular interest. Acetonitrile is one of the most widely and intensively used hydrophilic solvents in biotechnological and pharmaceutical industries. It is also involved in industrial chemical synthesis, formulation, concentration, extraction, product and by-product recovery and facilitation of waste-stream cleanup, and thus contributes significantly to high organic load in wastewater discharge. Biological treatment of acetonitrile using natural acetonitrile-degrading bacteria has been reported, but their remediation efficiency was limited due to low cell tolerance to the high toxicity of acetonitrile. Since there is increasing interest in engineering bacterial strains to employ in bioremediation process, bacteria with tolerance to solvents is highly desirable as a genetic host where specific biodegradation genes of the target pollutant is endowed (15).

**Fig. 2:**
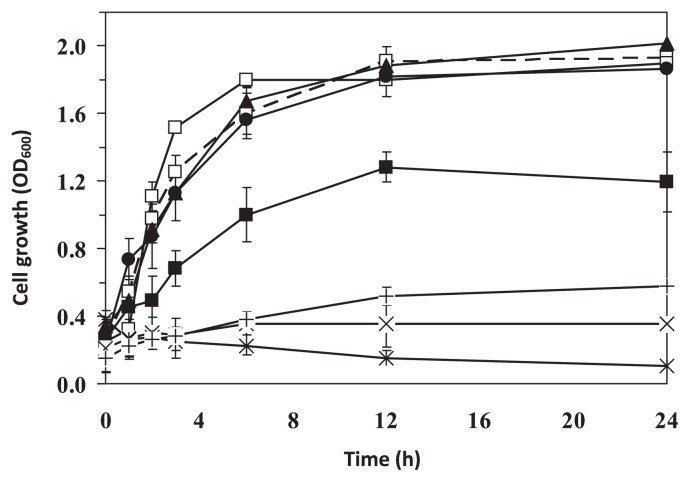
Acetonitrile tolerance of *Exiguobacterium* sp. SBH81 under culturing conditions in MSBYG medium. Cell growth was observed when acetonitrile was supplemented at the initial growth stage, as vapor phase (□) or as liquid (% [v/v]): 1 (▲), 3 (●), 5 (■), 6 (+), 7 (×) and 10 (*) (solid line). Cell growth on MSBYG medium in the absence of acetonitrile (□, dashed line) is shown as a control. Data are the means of the results of at least three individual experiments.

To apply a bacterium as a host for a bioremediation process or biotechnological fermentation, a cell’s ability to grow in the presence of solvent at its initial stage of growth is important and it was previously employed as a parameter to indicate the potential use of a bacterial strain as a host for the bioproduction of butanol (18); therefore, growth of SBH81 was examined when acetonitrile was added simultaneously with the bacterial inoculum. This test is different from the tolerance test method used during screening, where solvent was added to a suspension of high-density cells previously grown to the mid-exponential phase. The result showed that SBH81 was able to utilize acetonitrile vapor as sole carbon and nitrogen sources, but with a slow growth rate of 0.028 h^−1^ in MSB medium. Its tolerance was enhanced when grown in MSBYG medium to a higher concentration of acetonitrile (up to 3% [v/v]) (Fig. 2); however, it was adversely affected at 5% (v/v) and completely abolished at 10% (v/v) (Fig. 2). The higher solvent tolerance of SBH81 in glucose-containing medium or LB medium corroborates the fact that sugar and cell energy-providing nutrients could lead to an increase of energy supply and thus improve solvent tolerance. In addition, a recent study illustrated that yeast extracts directly supply and sustain bacterial biomass synthesis precursors, such as amino acids, therefore leading to the high solvent tolerance of bacteria (18).

There have been previous reports on bacteria able to tolerate and utilize acetonitrile under cell-growing conditions, such as *Natronocella acetinitrilica* gen. nov., sp. nov. (20), *Rhodococcus erythropolis*(1), *Comamonas* sp. (9); however, the acetonitrile concentration typically ranged from 0.8–24 mM due to its severe toxicity to cells (8). On the other hand, SBH81 was able to survive at a relatively higher concentration up to 5% (v/v) (950 mM); therefore, to our knowledge, this report is the first to describe a marine bacterium able to tolerate such a high concentration of acetonitrile under cell-growing conditions.

### Adaptation of SBH81 to acetonitrile

**Fig. 3:**
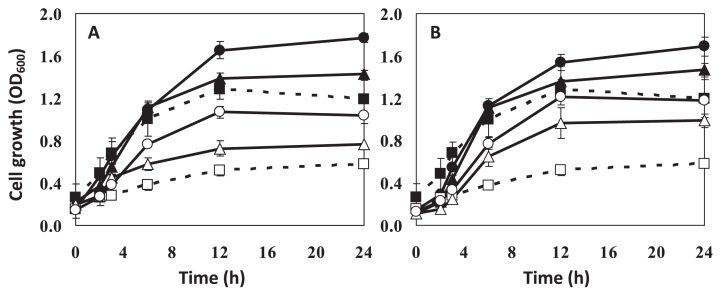
Acetonitrile tolerance of non-adapted (dashed line) and acetonitrile-adapted (solid line) *Exiguobacterium* sp. SBH81 grown on MSBYG medium. (A) Growth of non-adapted cells (square), 10-cycle acetonitrile-adapted cells (triangle) and 30-cycle acetonitrile-adapted cells (circle) was observed in the presence of acetonitrile: 5% (v/v) (■, ▲, ●, respectively) and 6% (v/v) (□, △, ○, respectively). (B) Tolerance stability of acetonitrile-adapted cell (expressed by growth in the presence of acetonitrile). The adapted cells were grown in MSBYG without acetonitrile for two growth cycles before re-exposure to acetonitrile at 5% (v/v) ((■, ▲, ●, respectively) and 6% (v/v) (□, △, ○, respectively). Data are the means of the results of at least three individual experiments.

Solvent tolerance of a host can be enhanced not only by modification of medium composition, but also by cell adaptation. To date, there have been a few reported adaptation approaches to improve bacterial solvent tolerance. One involves adaptation on an agar plate in a saturated solvent atmosphere (styrene). The second involves adaptation of growing cells in liquid medium with pulsed increase of solvent (toluene or styrene) (13); however, these strategies did not increase cell tolerance to hydrophilic solvent with high water solubility and toxicity (18). Therefore, a modified adaptation protocol was used in this study, where a constant level (a slightly toxic, but non-lethal level) of acetonitrile (4% [v/v]) was used in each batch of adaptation. The adapted cells exhibited significant improvement of tolerance as the biomass and growth rate were markedly increased in the presence of 5% (v/v) and 6% (v/v) acetonitrile (Fig. 3A). The increased growth rate of acetonitrile-adapted SBH81 may result from increased energy supply from acetonitrile and from changes in the metabolic responses of adapted cells (18).

Moreover, to examine whether the increase of cell tolerance was a stable characteristic, the adapted cells were grown in MSBYG medium without acetonitrile for two growth cycles, re-exposed to acetonitrile and then growth was evaluated (Fig. 3B). The adapted cells showed similar tolerance profile to the previous test (Fig. 3A), indicating that the adaptation characteristic was stably maintained. These results demonstrated that SBH81 has unique characteristics to cope with and stably adapt to a high concentration of acetonitrile.

### Tolerance mechanisms of SBH81 to acetonitrile

OST bacteria have developed resistance mechanisms in response to solvent exposure, of which three main mechanistic actions can be categorized: 1) prevention of solvent entry by modification of the cell membrane and cell morphological alteration; 2) solvent inactivation by solvent-degrading enzymes or solvent-emulsification agent; and 3) extrusion of solvents, after entering the cell, using an efflux system (19). While extensive studies on solvent tolerance mechanisms have been conducted in Gram-negative bacteria, little information on the solvent tolerance mechanisms of Gram-positive has been describe; therefore, mechanism studies were conducted in SBH81.

**Fig. 4:**
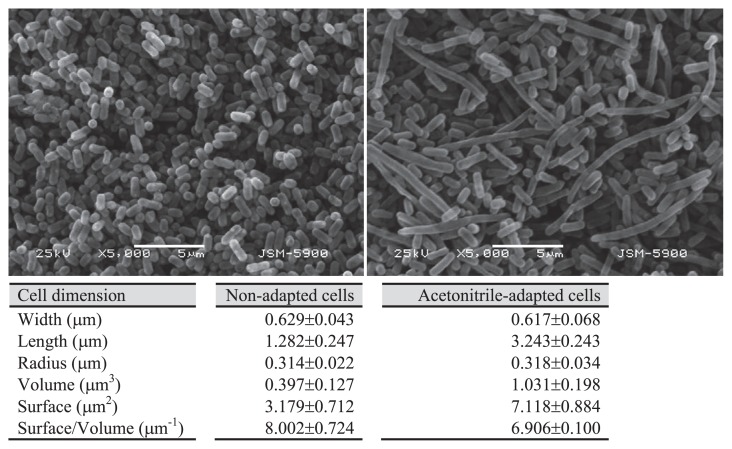
Morphology and dimension of *Exiguobacterium* sp. SBH81. Scanning electron micrographs show non-adapted cells (A) and 30-cycle acetonitrile-adapted cells (B). Cells were grown in MSBYG medium at 37°C for 24 h. Cell dimension was the average value of 150 individual cells, using SemAfore version 4.01 (JEOL, Sweden). Scale bars, 5 m. Magnification, ×5,000.

SBH81 is not a spore-forming bacterium; thus, protection from solvent by endospore formation did not occur. Changes in cell morphology and surface characteristics have been commonly described in Gram-negative bacteria as adaptive responses to environmental stresses. In Gram-positive bacteria that lack an outer membrane, the external layer of peptidoglycan plays an important role to oppose internal hydrostatic pressure, prevent membrane damage and allow cells to alter their morphology in response to solvent exposure (2). Using SEM analysis, non-adapted SBH81 was shown to have a short rod shape with a surface per volume (S/V) ratio of 8.002±0.724, whereas adapted cells increased their size (*i.e.* increased length with a constant radius) and a reduced S/V ratio to 6.906±0.100 (Fig. 4). The relative reduction of the cell S/V ratio as a response to acetonitrile exposure reduces the attachable cell surface to solvent, lowering solvent diffusion and partitioning into the cell membrane, therefore providing SBH81 higher tolerance to acetonitrile. A similar response was reported in Gram-negative *Enterobacter* sp. VKGH12 when exposed to butanol (13). In contrast, the opposite effect (*i.e.* reduction of cell size) was reported for cells under starvation stress (5) or stress from metal ions (16).

Modification of cell surface characteristics has been stated as another cell adaptation mechanism to solvent exposure (24). Nevertheless, the cell surface property of non-adapted and 30-cycle-adapted SBH81, examined using a BATH test, suggested a moderate hydrophobic cell surface with % hydrophobicity values of 49±7 and 48±6, respectively. This result suggested that modification of the cell surface may not contribute to solvent tolerance in SBH81.

It has been stated that solvent-degrading enzymes and solvent-emulsifying agents may take part in solvent inactivation to prevent cell damage. SBH81 could slightly degrade acetonitrile, indicating that it does not solely depend on acetonitrile degradation as the main tolerance mechanism (*i.e.* slight dissimilation of acetonitrile by SBH81 was inferred by 1) a slow cell growth rate of 0.028 h^−1^ in MSB medium supplemented with acetonitrile vapor, suggesting that cells could slightly assimilate acetonitrile for growth even at low concentrations, and 2) an insignificant change of the time course of acetonitrile degradation by SBH81 tested using gas chromatography analysis [data not shown]). In addition, cell supernatant, examined for solvent emulsification activity, did not show stable emulsion formation (data not shown), indicating that solvent-emulsifying agent may not be involved in the solvent tolerance of SBH81.

**Fig. 5:**
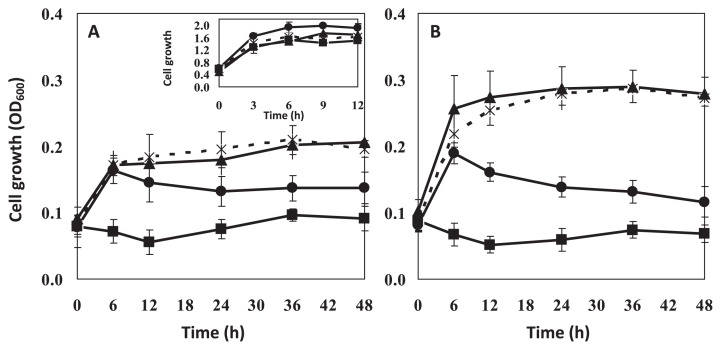
Effect of efflux pump inhibitor (EPI) to viability of *Exiguobacterium* sp. SBH81 in the presence of acetonitrile vapor. Non-adapted cells (A) and 30-cycle acetonitrile-adapted cells (B) were grown in MSB medium supplemented with acetonitrile vapor without EPI (*) and with EPI: PAβN (0.1 mM) (■), paroxitine (0.1 mM) (●), and orthovanadate (10 mM) (▲). The initial cell OD_600_ at approximately 0.19 and that at the end point at approximately 0.11 corresponded to the value ranging from 10×10^6^ and 5×10^6^ CFU mL^−1^, respectively. Effect of EPI on cell growth in MSBYG medium in the absence of acetonitrile is shown in the inset. Data are the means of the results of at least six individual experiments.

Solvent tolerance mechanisms examined in SBH81, as mentioned above, involved bacterial protection mechanisms that diminish solvent entry into cells. In addition, bacteria also have an energy-dependent efflux system as a major defensive mechanism to extrude solvents that have entered the cell. The majority of efflux pumps identified in Gram-negative bacteria for solvents belong to the RND family (a secondary transporter which utilizes the proton gradient as a source of energy for solvent secretion) (3, 10, 17); however, in Gram-positive bacteria, the RND family and ATP-binding cassette (ABC) transporter were reported (11). The involvement of an efflux system in the solvent tolerance of SBH81 was examined by determining the effect of a specific efflux pump inhibitor (EPI) on cell viability in MSB medium in the presence of acetonitrile vapor. The EPIs used in this study were as follows: (1) orthovanadate, an inhibitor of the ABC family (11); (2) paroxitine, RND inhibitor; (3) PAβN, an inhibitor of RND and the multidrug and toxic compound extrusion (MATE) family (10). The EPI concentration used had no effect on cell viability when grown on MSBYG (Fig. 5A, inset); however, in the presence of acetonitrile, cell viability was adversely affected by the presence of paroxitine and PAβN (Fig. 5A and B), indicating that the secondary transporters, RND and MATE family efflux systems, may be involved in acetonitrile tolerance in SBH81. In the absence of the inhibitor, acetonitrile taken up into cells was partly utilized for cell growth and partly pumped out by the active efflux system. In the presence of paroxitine and PAβN, acetonitrile was not pumped out and its accumulation inside cells led to decreased cell viability and, eventually, to cell death. On the other hand, the addition of orthovanadate did not affect cell viability in the presence of acetonitrile (Fig. 5A and B), indicating that an ABC-type transporter may not be required for acetonitrile tolerance in SBH81. In Gram-positive bacteria, which contain a single membrane envelope, the existence of an active efflux system is necessarily important as a defensive mechanism of toxic solvents; therefore, the presence of multiple efflux pumps/transporters with overlapping substrate specificity has been generally reported in one organism (10). SBH81 may possess a variety of efflux pumps; nonetheless, in this study, we report the involvement of RND and MATE family efflux systems in its acetonitrile tolerance.

In conclusion, *Exiguobacterium* sp. SBH81 was shown to have high tolerance to various types of toxic hydrophilic solvents. It exhibited tolerance to acetonitrile at relatively high concentrations, *i.e.* up to 10% (v/v) by resting cells (Table 1) and up to 6% (v/v) under growing-culture conditions (Fig. 2). Its tolerance mechanisms rely on morphological changes and the secondary transporter efflux system. The fact that SBH81 has high solvent tolerance under resting cell and culturing conditions, and is susceptible to solvent tolerance enhancement, make it attractive as a genetic host for biotechnological production and bioremediation applications.

## Figures and Tables

**Table 1:** Organic solvent tolerance of *Exiguobacterium* sp. SBH81

Organic solvents	Log *P*_ow_^a^	Cell survival	

		Organic solvent at 5% (v/v)	Organic solvent at 10% (v/v)
Decane	5.01	+++	+++
Heptane	4.66	+++	+++
Octane	5.18	+++	+++
Decanol	4.23	+	+
Hexane	3.90	+	+
Cyclohexane	3.44	+	+
Xylene	3.20	−	ND
Ethylbenzene	3.15	−	ND
Styrene	2.95	−	ND
Toluene	2.73	−	ND
Heptanol	2.62	−	ND
Diethylphathalate	2.42	+++	+++
Benzene	2.13	++	++
Chloroform	2.00	−	ND
Butyl acetate	1.78	−	ND
Pentanol	1.51	−	ND
Diethyl ether	0.89	+++	ND
Butyraldehyde	0.88	−	ND
Butanol	0.88	++	−
Ethyl acetate	0.73	−	ND
Propionaldehyde	0.59	+	ND
Propanol	0.25	++++	+++
Ethanol	−0.31	+++	+++
Acetonitrile	−0.34	+++	+++
Methanol	−0.77	+++++	+++++
Dimethylformamide	−0.87	+++++	++++
Dimethyl sulfoxide	−1.35	+++++	+++++

Cells were initially grown to the mid-exponential phase in MSBYG medium before organic solvent was directly added. Cell survival was examined after 12 h of solvent exposure. The number of surviving cells is represented by symbols: − (no survival) and + (survival). The number of plus signs corresponds to cell numbers (CFU mL^−1^): + (<10^2^), ++ (10^2^–10^4^), +++ (10_4_ to 10^6^), ++++ (1×10^8^–50×10^8^), +++++ (50×10^8^–100×10^8^), and ND (not determined).

a Log *P*_ow_ values were according to K_owwin_ version 1.67, EPI Suite (US Environmental Protection Agency).
